# Separate Effects of Sodium on Germination in Saline–Sodic and Alkaline Forms at Different Concentrations

**DOI:** 10.3390/plants12061234

**Published:** 2023-03-08

**Authors:** Yasmeen Hitti, Sarah MacPherson, Mark Lefsrud

**Affiliations:** Department of Bioresource Engineering, McGill University, 21111 Lakeshore Road, Sainte-Anne-de-Bellevue, QC H9X 3V9, Canada

**Keywords:** germination, alkalinity, sodicity, salt stress, hydroponic experiment

## Abstract

Salinity negatively impacts crop productivity, yet neutral and alkali salt stresses are not often differentiated. To investigate these abiotic stresses separately, saline and alkaline solutions with identical concentrations of sodium (12 mM, 24 mM and 49 mM) were used to compare the seed germination, viability and biomass of four crop species. Commercial buffers containing NaOH were diluted to generate alkaline solutions. The sodic solutions tested contained the neutral salt NaCl. Romaine lettuce, tomato, beet, and radish were seeded and grown hydroponically for 14 days. A rapid germination was observed for alkaline solutions when compared to saline–sodic solutions. The highest plant viability recorded (90.0%) was for the alkaline solution, containing 12 mM Na^+^, and for the control treatment. Plant viability, with a value of 49 mM Na^+^ in saline–sodic and alkaline solutions, was the lowest (50.0% and 40.8% respectively), and tomato plants did not germinate. EC values were higher for the saline–sodic solutions than the alkaline solutions, yielding greater fresh mass per plant for all species, with the exception of beets grown in alkaline solution, with a value of 24 mM Na^+^. The fresh mass of romaine lettuce grown in the 24 mM Na^+^ saline–sodic solution was significantly greater than romaine lettuce grown in the alkaline solution with the same sodium concentration.

## 1. Introduction

Plants are often subjected to unfavourable growth conditions, including high soil salinity and alkalinity, arising from natural or man-made causes [1]. Salinity is mainly caused by NaCl accumulation, while alkalinity is caused by the accumulation of NaHCO_3_ and Na_2_CO_3_ [2]. Salinity can inhibit and delay plant germination, growth, and development [3,4], and salt stress is most often described as the effects of NaCl, or sodicity, on plant growth. Nearly all alkaline environments contain salts. However, sodicity and alkalinity effects are often grouped together, and limited information differentiates or distinguishes between the separate effects of neutral and alkali salts on plant growth [5,6,7]. 

Salinity may be defined as the proportion of incorporated salts in a solution, while alkalinity is defined as the degree of basicity of a solution [8]. Typical saline cations are Na^+^, Ca^2+^, K^+^ and Mg^2+^. These cations are commonly paired with HCO_3_^−^, Cl^−^, and SO_4_^2−^ to create neutral salts [1]. In an aqueous solution, these cations are exchangeable, and can pair with different hydroxides [9], including OH^−^ and CO_3_^−^, to form alkali salts [10]. Available ion concentrations in water or soil can have detrimental saline or alkaline consequences on plant growth [3]. In addition, nutrient availability is obstructed in high-pH solutions or media [11] and the preferred pH range for plant growth is 6.5 to 7.5 [12].

Previous experiments have used varied concentrations of major macronutrients in the treatment of plants, in order to investigate their adverse effects [13]. Although the influence of certain elements, including salts, on plant germination, growth, and development has been well studied, studies investigating the impact of separate saline and alkaline anions or cations are lacking. Measuring the specific effect of alkaline stress presents a challenge, as a plant’s exposure to alkaline and saline stress normally occurs together [14]. It is similarly difficult to study the effects of pH on plant growth, as plant nutrient solutions consist of an ion equilibrium, and the separate effects of hydrogen and hydroxyl groups on plant growth are not often explored [13].

A growing global awareness of soil salinity and alkalinity as an environmental threat and serious crop production problem has prompted several reports that have attempted to understand the separate effects of sodicity and alkalinity on seed germination and plant growth [7,15,16]. With this study, we contribute to this body of knowledge by separating and testing the impact of sodicity (NaCl) and alkalinity (NaOH) on the germination, early seedling viability and biomass production of four food crop plant species: *Lactuca sativa* (romaine lettuce), *Solanum lycopersicum* (tomato), *Beta vulgaris* (beet), and *Raphanus raphanistrum* (radish). The study provides insight into saline–sodic and alkaline stresses in hydroponic solutions and how they differentially affect plant species. 

## 2. Materials and Methods

### 2.1. Test Solutions

Alkaline solutions were derived from the commercial buffer solutions listed in Table 1, and these provided a constant pH throughout the experiment. The saline–sodic and alkaline solutions, comprising different [Na^+^] concentrations that were tested, are listed in Table 2. The sodium concentration [Na^+^] of each commercial buffer was pre-determined by the manufacturer. The pH 8 buffer was diluted with water, at 1: 4 and a 1: 2 ratios, to yield low-NaOH and mid-NaOH solutions, respectively. A 1: 2 dilution of the pH 9 buffer with dH_2_0 yielded the high-NaOH solution. Corresponding concentrations of NaCl (0.72 g/L, 1.43 g/L, and 2.87 g/L) were diluted in dH_2_0 to yield low-NaCl (12.32 mM Na^+^), mid-NaCl (24.47 mM Na^+^), and high-NaCl (49.11) solutions.

### 2.2. Germination and Plant Viability

The germination of four plant species was tested in control (distilled H_2_O), saline–sodic, and alkaline solutions, with increasing sodium concentrations (Table 2). A total of 32.4 cm^2^ rockwool cubes (Grodan A-OK Starter Plugs, The ROCKWOOL Group) were soaked in each solution, and then seeded with four different plant species (8 cubes per species): romaine lettuce, tomato, beet, and radish. Seeded cubes were placed in a tray in a growth chamber (TC30, Conviron, Canada) with pre-set conditions: 25 °C, 50% relative humidity, and a 16 h photoperiod. The lighting in the growth chamber consisted of fluorescent lights, and a light intensity of 150 µmol m^−2^s^−1^ was measured at the base of the cubes. Germinated seeds were counted every 2 days and the germination percentage was defined as the number of successfully germinated seeds over the total number of sown seeds. Solutions were replenished twice throughout the experiment to an equal volume and concentration. The germination test was replicated three times.

### 2.3. pH

The pH of each solution was measured at 2 day intervals for the duration of the experiment. A hand-held pH probe (Accumet AB15, Fischer Scientific, Waltham, MA, USA) was placed in the hydroponic tray in three random locations, and the mean was calculated.

### 2.4. Electrical Conductivity (EC)

The EC of each solution was measured at 2 day intervals for the length of the experiment. A hand-held EC probe (DiST 6 EC/TDS/Temperature Tester, Hanna Instruments, Smithfield, RI, USA) was placed in the hydroponic tray in three random locations, and the mean was calculated.

### 2.5. Fresh Mass Yield

At day 14, the fresh shoot biomass was measured for all plants by using a scale (APX-153, Denver Instruments, NY, USA). The plant fresh mass was low for all species, and the amount of dry mass was too low to measure accurately with this instrument.

### 2.6. Statistical Analysis

To compare fresh mass yields amongst the different plant species investigated, a one-way ANOVA was performed using Rstudio (Integrated Development Environment for R; Boston, MA, USA). A post-hoc analysis, Tukey’s Honest Significant Difference (Tukey HSD), was used to compare treatments at a 95% confidence interval. The normality of the data and the homogeneity of variances were verified by performing the Shapiro–Wilk and Levene tests, respectively.

## 3. Results

To determine the separate effects of sodicity and alkalinity on plant germination, alkaline and saline–sodic solutions with increasing concentrations of Na^+^ were generated (Table 2). At day 0, seeds from the four different species, romaine lettuce, tomato, beet, and radish, were sown into rockwool cubes soaked in each test solution. EC, pH values, germination percentages and plant viability were recorded at 2 day intervals, while the fresh mass yield of each species was measured after the 14 day growth period.

### 3.1. EC

**Figure 1:**
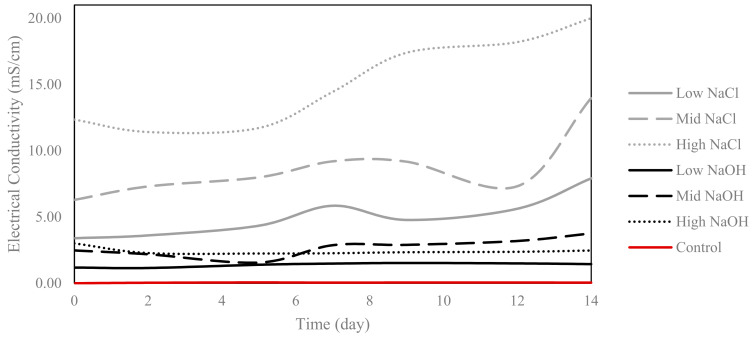
Recorded electrical conductivity (EC) of the saline–sodic and alkaline solutions over the 14 day period.

All saline–sodic (NaCl) solutions maintained higher conductivity than the alkaline solutions for the duration of the experiment (Figure 1). As expected, the highest EC value corresponded to the high-NaCl solution; at day 0 it was 12.35 mS/cm and at day 14 it was 20.00 mS/cm. This last value (20.00 mS/cm) was equal or greater to the maximal range of the EC sensor. EC values for the low-NaCl solution were 3.42 mS/cm and 7.89 mS/cm at days 0 and 14, respectively. For the mid-NaCl solution, EC values were 6.27 mS/cm and 14.43 mS/cm at days 0 and 14, respectively.

The EC of the alkaline solutions did not increase extensively over the 14 day period. The mid-NaOH solution experienced a drop in EC at day 5, and surpassed the EC of the high-NaOH solution after day 6. In addition, the highest EC recorded for the alkaline solutions was for the mid-NaOH solution, at day 14 (3.88 mS/cm). At day 0, the EC of the low-NaOH solution was 1.2 mS/cm, and at day 14 the final EC was 1.72 mS/cm. The high-NaOH solution decreased in terms of the EC over the 14 day period; at day 0, the EC was 2.98 mS/cm, and at day 14 it was 2.34 mS/cm. As expected, the control solution had the lowest EC, remaining constant at ~0.04 mS/cm for the duration of the experiment.

### 3.2. pH

**Figure 2:**
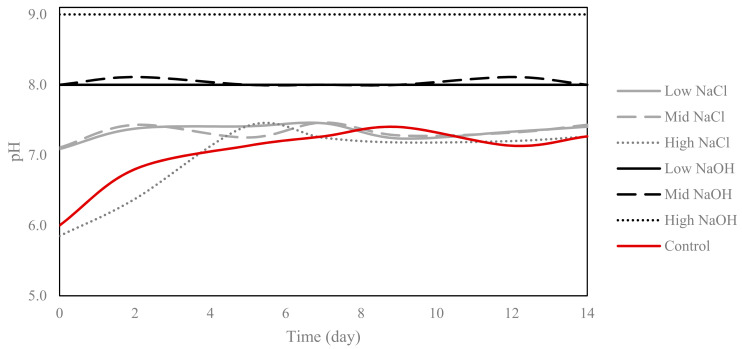
Measured pH values of the saline–sodic and alkaline solutions over the 14 day period.

The pH of all alkaline solutions remained constant, and exceeded the pH values of all saline–sodic solutions tested over the 14 day period (Figure 2). As expected, a pH value of approximately 8 was measured for the low-NaOH solution throughout the study. The pH of the mid-NaOH solution fluctuated slightly at days 2 and 12. The pH of the high-NaOH solution (½ pH 9 buffer) was ~9 throughout the 14-day period. The pH of the high-NaCl solution was the lowest among all solutions until day 4; at day 0, it was 5.8, and at day 4 it was 6.3. The low-NaCl and mid-NaCl solutions had similar pH values for the duration of the experiment, fluctuating between 6.9 and 7.4. Similar pH values were recorded for all saline–sodic solutions from days 6 to 14. The pH of the control was 5.3 at day 0 and was 7.4 at day 14.

### 3.3. Germination and Plant Viability

**Figure 3:**
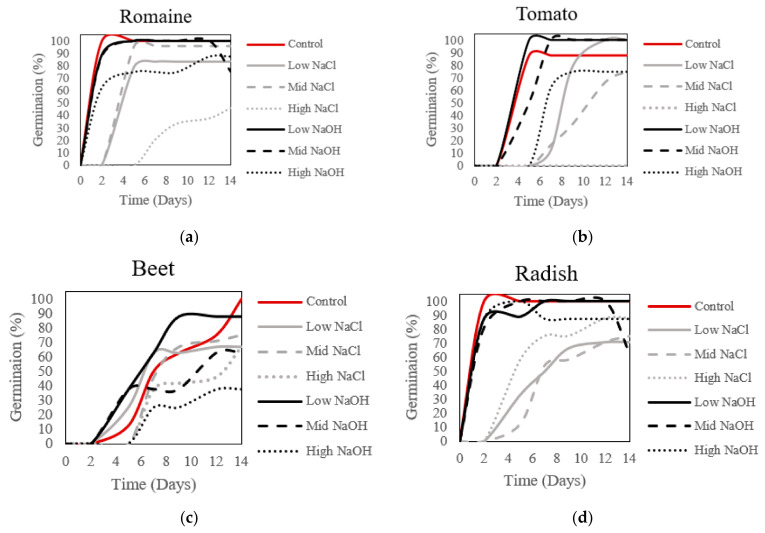
Germination and viability of plants grown in saline–sodic and alkaline solutions for 14 days. (**a**) Viability of romaine plants, with alkaline solutions having a higher success rate than sodic solutions. (**b**) Viability of tomato plants with alkaline and low-sodic solutions, with a higher success rate. (**c**) Beet plants with lower alkaline solutions and control solutions had increased viability. (**d**) Radish plants with alkaline solutions had increased viability.

To determine the influence of saline–sodic or alkaline salt stress on seed germination and plant viability, germination of the four experimental plant species was recorded for the seeds sown in the saline–sodic or alkaline solutions with different concentrations of sodium over the 14 day period (Figure 3). Germination percentage and plant viability were defined as the percentage of successfully germinated seeds over the total number of planted seeds for each solution. The highest plant viability recorded was for the control solution, with the exception of tomato seedlings, where the low-NaOH solution yielded a higher success rate. For romaine, tomato, and radish, germination rates from day 0 to 12 were higher in the low- and mid-NaOH solutions. For these three species, the low- and mid-NaOH solutions did not seem to hinder the germination time as much as the NaCl solutions. A two day delay in germination could be seen for the NaOH solutions, between the romaine and radish plants. As for tomato seedlings, a 4 day delay could be seen, between low and mid-NaOH and low- and mid-NaCl groups. Beet plants exhibited a higher germination rate with the low NaOH solution, and this was followed by the rates achieved with the low- and mid-NaCl solutions, which differed from the romaine, tomato and radish germination curves. After 14 days, plant viability for seeds sown in the low-NaOH and control solutions was approximately 100.0%, with the exception of beets, at 87.5%. Plant viability for seeds sown in the mid-NaOH solution declined from days 12 to 14 for romaine and radish; germination rates dropped from 100.0%, to 75.0% and 62.5%, respectively. High-NaOH and high-NaCl solutions exhibited greater plant viability for radish plants, and lower concentrations of NaCl in the radishes were not as successful. Tomato was the only species to not germinate at all in high-NaCl solutions.

### 3.4. Fresh Mass Yield

**Figure 4:**
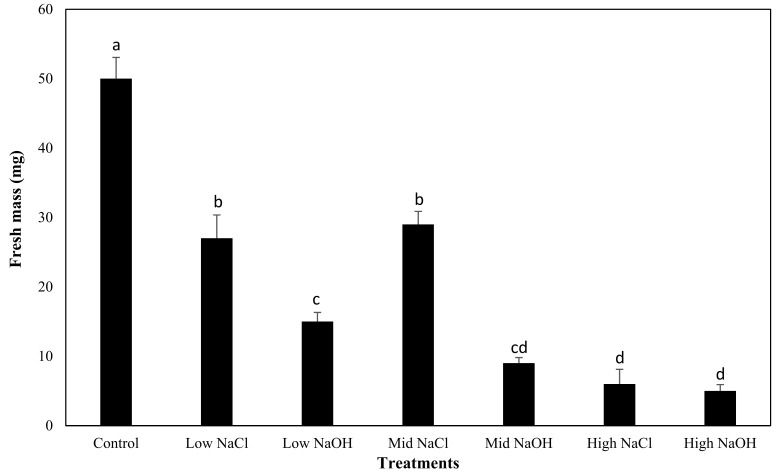
Comparison of the mean fresh mass yield of romaine lettuce plants grown for all treatments and their respective standard errors. The characters represent the different groups found through the Tukey’s test.

To determine if biomass was influenced by saline–sodic or alkaline salt stress, fresh mass yield of plants grown in the different solutions was compared for romaine lettuce, tomato, beet, and radish. The fresh mass yield of romaine lettuce plants grown in water (control) was greatest (0.050 g per romaine lettuce plant), and was significantly higher than romaine lettuce plants grown under saline–sodic or alkaline salt stress (Figure 4); romaine lettuce plants grown in sodic–saline solutions had a higher fresh mass yield than romaine lettuce grown in alkaline solutions. However, this difference was only significant between romaine lettuce plants grown in 24.47 mM Na^+^ (mid-NaCl) and lettuce grown in an alkaline solution, with the same amount of sodium (mid-NaOH). No significant difference in fresh mass yield between saline–sodic and alkaline solutions containing 49.11 mM NaCl was observed. The lowest fresh mass yields were recorded for plants grown in saline–sodic and alkaline solutions at 49.11 mM Na^+^, and these values were similar (high-NaOH (0.005 g per plant) and high-NaCl (0.006 g per plant); group d, *p* > 0.05).

**Figure 5:**
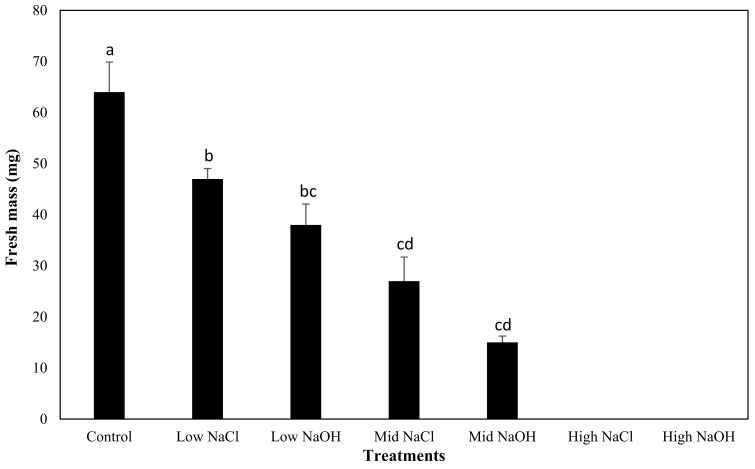
Comparison of the mean fresh mass yield of tomato plants grown in all the treatments, and their respective standard errors. The characters next to the bars represent the different groups found through the Tukey’s test.

As expected, the mean fresh mass yield of tomato plants was greatest when grown in water (control; 0.064 g per plant), and this was significantly higher than tomato plants grown in any of the saline–sodic or alkaline solutions (group a; Figure 5). While the fresh mass yield of the tomato plants appeared to be higher than in the saline–sodic solutions when compared to alkaline solutions, statistical analyses showed no significant differences in fresh mass yield between tomato plants grown in saline–sodic or alkaline solutions, with a value of 12.2 mM Na^+^ (low-NaCl and low-NaOH; group b, *p* > 0.05) and 24.47 mM Na^+^ (mid-NaCl vs. mid-NaOH solution; group d, *p* > 0.05), respectively. No tomato plants germinated in the high sodium solutions, regardless of sodicity or alkalinity.

**Figure 6:**
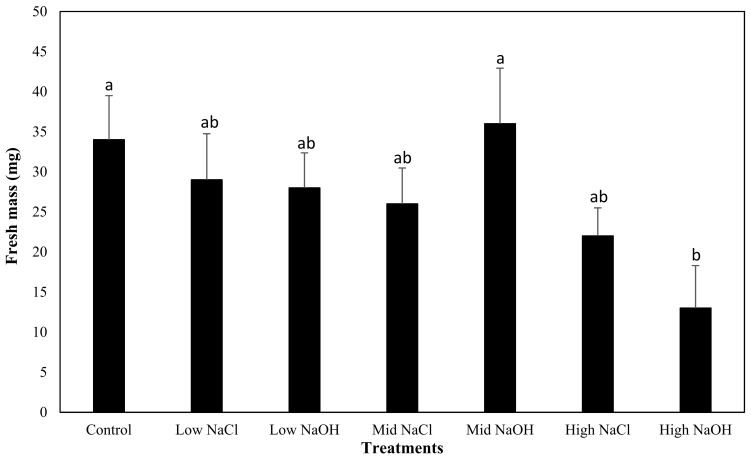
Comparison of the mean fresh mass yield of beet plants grown for all treatments, and their respective standard errors. The characters next to the bars represent the different groups found through the Tukey’s test.

For beet plants grown in different saline–sodic or alkaline solutions, the highest mean fresh mass yield was observed when plants were grown in an alkaline solution, with a value of 24.47 mM Na^+^ (mid-NaOH; 0.036 g per beet plant), but this was not significantly greater when compared to beet plants grown in the control (group a: Figure 6). This fresh mass yield was also greater than plants grown in the saline–sodic solution, with a value of 24.47 mM Na^+^, but at high sodium concentrations, fresh mass yield was greater in the saline–sodic solution than in the alkaline solution. The lowest mean fresh mass yield was measured in beet plants grown in the high-NaOH solution (0.013 g per beet plant).

**Figure 7:**
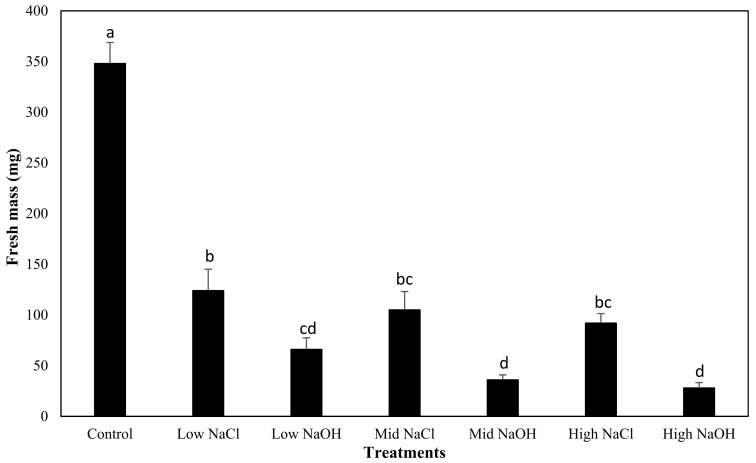
Comparison of the mean fresh mass yield of radish plants grown for all treatments, and their respective standard errors. The characters next to the bars represent the different groups found through the Tukey’s test.

The mean fresh mass yield of radish plants was greatest when grown in water (control; 0.348 ± 0.102 g per plant, group a), and was significantly higher than beet plants grown in any of the saline–sodic or alkaline solutions with different concentrations of sodium (Figure 7; group a, *p* > 0.05). Importantly, fresh mass was higher for radish plants grown in saline–sodic solutions when compared to alkaline solutions with the same sodium concentrations. Sodium concentration had no significant effect on mean fresh mass yield for all three saline–sodic solutions (low-NaCl, mid-NaCl, and high-NaCl; group b, *p* > 0.05) or all three alkaline solutions (low-NaOH, mid-NaOH, and high-NaOH; group d). Similarly, there were no significant differences in mean fresh mass yield for radish plants grown in the saline–sodic mid-NaCl and high-NaCl solutions, or in terms of the alkaline, low-NaOH solution (group c). The lowest fresh mass yield was observed in radish plants grown in the high-NaOH solution (0.028 g per radish plant).

## 4. Discussion

The recommended EC of a nutrient solution for optimal plant growth in a hydroponic system is between 0.8 and 3.7 mS/cm [17]. In this study, only the control and alkaline solutions fell within this recommended EC range for plant growth, while the saline–sodic solutions exceeded this range. When comparing the tested alkaline and saline–sodic solutions, EC values were higher for the saline–sodic solutions than the alkaline solutions, and solutions with higher EC yielded larger quantities of fresh biomass per plant for all species, except for the beets grown in the mid-NaOH solution. According to the literature, solutions with elevated EC imply that the system is ion-rich, and this indicates ion mobility [18,19]. Furthermore, conditions with high EC can cause stress to growing plants, as a high EC limits the amount of free water [20]. Neutral and alkaline solutions typically have an elevated EC, yet the nature of the salt ions will dictate if the overall pH of a solution will be affected [3,4]. As the saline–sodic and alkaline solutions had identical concentrations of sodium, the high EC can be directly associated with the chlorine anion [21].

While the low-NaOH and mid-NaOH alkaline solutions originated from the same buffer and had similar pH values of approximately eight, EC values varied slightly. The high-NaOH solution had a lower EC than the mid-NaOH solution, suggesting that other elements in the commercial buffer were more conductive. Of the saline–sodic solutions, lower pH values were recorded in the high-NaCl solution, which also had an elevated EC. Regulation of the nutrient solution’s pH in hydroponic systems has been linked to a plant’s ability to uptake nutrients and to grow healthily [22,23]. Notably, the literature states that the recommended pH range for hydroponic solutions to allow for the availability of all nutrients is between 5.5 and 5.8 [24].

Past studies have concluded that seed germination and seedling establishment are the most critical stages for plant survival in a saline–alkaline environment [25]. High salinity delays, and can inhibit, seed germination [26], while increases in pH reduce plant growth [27]. In this study, the plant viability of seeds sown in the low-NaOH solution surpassed the plant viability of the control treatment; the solution had a pH of eight, which exceeds the advised pH range for hydroponic systems. In addition, plants subjected to alkaline salt stress demonstrated higher plant viability than those subjected to saline–sodic salt solutions at the sodium concentrations tested (~12–49 mM Na^+^). Specifically, the low-NaOH solution exhibited greater plant viability than seeds sown in the low-NaCl solution, and plant viability of the high-NaOH solution surpassed that of the high-NaCl solution by approximately 10% for the first 7 days of the experiment (Figure 3). In ryegrass, no significant effects on germination were noted when seeds were subjected to neutral salt or alkaline salt stress at 50 mM Na^+^ [26], whereas low Na^+^ concentrations in high pH environments can increase the speed at which sorghum seeds germinate [28]. While tomato seeds in this study did not germinate after 14 days at 49.11 mM Na^+^, it may be worthwhile to extend the growing period and widen the range of sodium concentrations used to grow these plant species, in order to determine if germination is simply delayed, and at what point seedling viability is affected.

Of course, differences in seed, seedling, and plant tolerance to salt stress between species is considerable, and should not be dismissed. The data reported herein demonstrate that the effects of neutral and alkaline stress on fresh mass yield are plant-species-dependent. Romaine, tomato, and radish plants had higher fresh mass yields when grown in the saline–sodic solutions than when grown in the alkaline solutions. For beet plants, however, the highest fresh mass per plant was observed when grown in the mid-NaOH solution. Beet plants are considered to be particularly resistant to salts; however, most plants are negatively impacted by salts in their germination period, this explains the low amount of fresh biomass produced [3,29,30]. In the seedling phase, however, beets are susceptible to salinity and not as tolerant to salts [31]. The beet plants in this experiment were not as affected by sodium in either saline–sodic or alkaline form in comparison with the other plants tested in this experiment, and supports findings from similar experiments done on seedlings by Geng et al. [32].

Significant differences (*p* < 0.05) in fresh mass yield between the low-NaOH and mid-NaOH solutions were observed for radish and tomato, suggesting that increases in sodium and hydroxide are more detrimental to some plant species’ growth than increases in sodium and chlorine. However, the pH for both of these solutions was eight, suggesting that pH may be independent of the negative effects of sodium, as well as the EC of a nutrient solution, on plant growth.

Previous research has reported that saline environments give rise to limited water availability, ion excesses, and decreases in the absorption of essential nutrients [6,33]. In addition, the absorption is reduced in alkaline and elevated pH environments [11,34,35]. Ambiguity takes place when discussing the hydroxyl ion concentration and its effects on plant physiology [36]. When there are salts present, an environment is most likely saline and alkaline, yet the combined effects of both properties remain obscure. Even in this study, studying the isolated separate effects of neutral and alkali salts is difficult, since the commercial buffers used for the experiment to make the alkaline solutions had alkali and neutral salts that contained sodium, while the saline–sodic solution contained only one neutral salt (NaCl). Problematic nutrient uptake can be caused by ionic concentrations and the pH of the solution; consequently, it can negatively impact the development and growth of plants. Both types of effects were observed in the saline–sodic and alkaline solutions investigated.

Evidence of nitrogen deficiencies was observed for plants grown in the high-NaOH solution in the form of chlorosis, with discoloration in the seedling leaves. Beet seedlings had bright pink leaves, whereas romaine lettuce and radish displayed yellow leaves. Chlorosis is a common indicator of high alkalinity [11,37]. When grown in the saline–sodic solutions, no physiological signs and no apparent changes in colour for any plant species were observed with any sodium concentration investigated; however, harmful effects were observed for tomatoes grown in the high-NaCl solution. However, the saline–sodic solutions provided a neutral pH environment and did not contain any other elements such as phosphate, potassium, and borates that were found in the alkaline solutions derived from commercial buffers. As such, the different pigmentation, slow development and small fresh mass observed in these plants under alkaline salt stress could be due to the elements in the buffer solution and the salt concentrations at which the plants were exposed. All buffers used had potassium present, which was likely absorbed by plant roots as K^+^ cations [38]. Sodium was present in the pH 8 and pH 9 buffer solutions and in all the saline–sodic solutions tested. Plant roots absorb Na^+^ cations and boron (present in the high-NaOH solution) in the form of BO_3_^2−^ anions [38].

Sodium and potassium are alkali metals that can form, respectively, neutral and alkali salts, depending on the anion they react with [39,40]. The literature suggests that stresses from alkaline and neutral salts are two separate types of abiotic stressors [34,41,42,43,44]. Sodium in the pH 8 and pH 9 buffers was paired with a hydroxide (NaOH). In these solutions, sodium is an alkali and its negative effects on plant growth are associated with alkali stress [5]. In the saline–sodic solutions, sodium is paired with a chloride anion (NaCl), and this results in the formation of a neutral salt. Potassium is paired with different anions in the buffer solutions: KH_2_PO_4_ in the pH 8 buffer and KCl in pH 9 buffer. As seen with sodium, this alkali metal may act differently when paired with a different anion. In this experiment, potassium’s interactions with sodium still remain unclear, as potassium can be found as a neutral, alkali or alkaline–saline salt.

Salt stress and the ratio of sodium to potassium cations can influence the turgor pressure of plant cells and drive osmosis [45]. In a healthy plant, Na^+^ is present in low concentrations in the cytoplasm, along with elevated amounts of K^+^ [6]. EC increases as the alkalinity of buffer solutions increases, and as EC increases, the concentration of salts increase, resulting in elevated osmotic potential [46]. Alkali stress exerts the same stress factors as salt stress, but with the addition of high-pH stress [44]. Alkalinity can be prevented with the addition of specific ions, and some ions can stop the negative effect of high salinity and alkalinity in these types of growth environments [45]. Salts can further impact plant growth when present in solutions at higher concentrations than when present in solutions that are high in alkalinity and low in salinity; the effects of a low alkali–saline concentration in solution can be similar to a high-neutral saline–sodic concentration in solution [5]. Interestingly, our data contradict previous findings that the low uptake of nutrients and unfavourable plant development are caused solely by high pH in plant germination experiments [27,47,48]. We theorise that the development of new nutrient solutions that counteract alkaline stress and will allow for timely germination and plant growth in unfavourable environments is possible.

## 5. Conclusions

Romaine lettuce, tomato, beet and radish plant growth is hindered when sown in hydroponic solutions with higher pH, elevated EC, and at high concentrations of sodium. Alkaline hydroponic solutions negatively impacted most plant growth more than sodic stress at certain sodium concentrations, whereas beet plants grow better in alkaline conditions than in saline-sodic solutions with identical concentrations of Na^+^. These results support previous studies demonstrating that saline-sodic and alkaline stresses exert different effects on plant growth. This study highlights that these influences are species-specific and that testing in hydroponic systems can be useful for studying the impacts of specific ions. Further studies are needed to understand the impact that different salt and alkali ions can have on desired crop growth in hydroponic systems.

## Figures and Tables

**Table 1:** Commercial pH 8 and pH 9 buffer components.

Buffer	Components	Manufacturer
pH 8	KH_2_PO_4_, NaOH	Thermo Fisher Scientific (Portsmouth, NH, US)
pH 9	H_2_O, KCl, H_2_BO_3_, NaOH, and preservative	Ricca Chemical Company (Arlington, TX, US)

**Table 2:** Saline–sodic and alkaline solutions tested to compare germination, seedling viability and fresh biomass.

		Saline-Sodic			Alkaline		
Solution	Control	Low-NaCl	Mid-NaCl	High-NaCl	Low-NaOH	Mid-NaOH	High-NaOH
NaCl [mM]	-	12.32	24.47	49.11	12.32	24.47	49.11
Buffer volume	-	-	-	-	¼ pH 8 buffer	½ pH 8 buffer	½ pH 9 buffer

## Data Availability

The data presented in this study are available on request from the corresponding author. The data are not publicly available due to confidentiality.
